# Supplementary material for: Exploring Charged Polymeric Cyclodextrins for Biomedical Applications
Source: Molecules. 2021 Mar 19;26(6):1724. doi: 10.3390/molecules26061724 (PMC8003440; doi:10.3390/molecules26061724)
Supplement: Supplementary file 1 [file molecules-26-01724-s001.zip › molecules-1128851-supplementary.pdf]

Supplementary Data

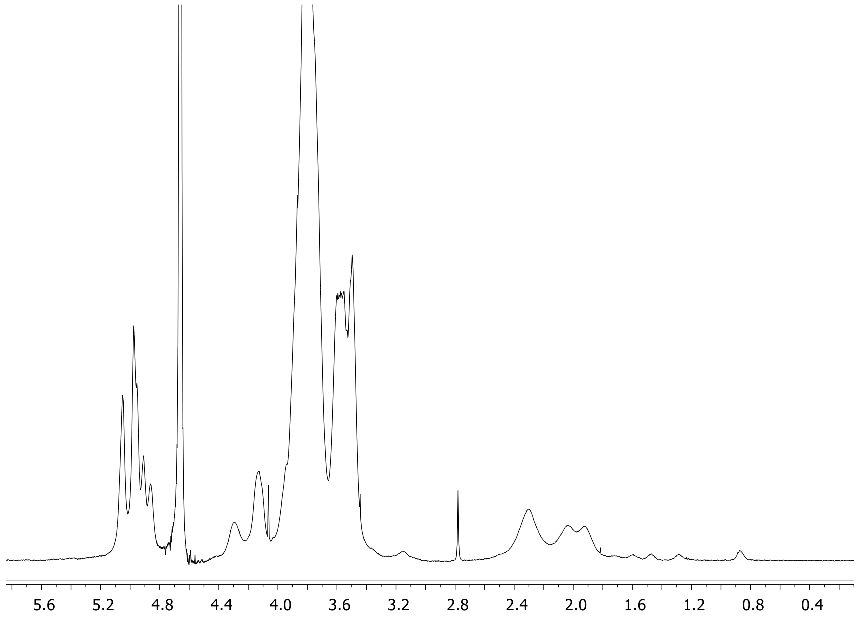

Figure S1. *^{1}H NMR spectrum of PGAβCyDArg1 (D_2O, 500 MHz)*

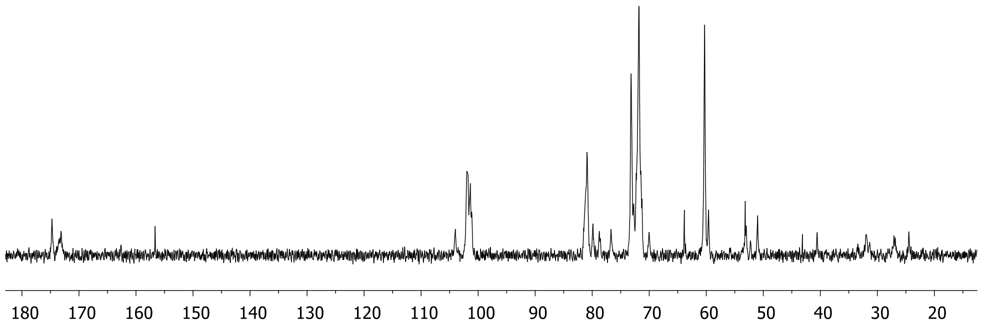

Figure S2. *^{13}C NMR spectrum of PGAβCyDArg1 (D_2O, 125 MHz)*

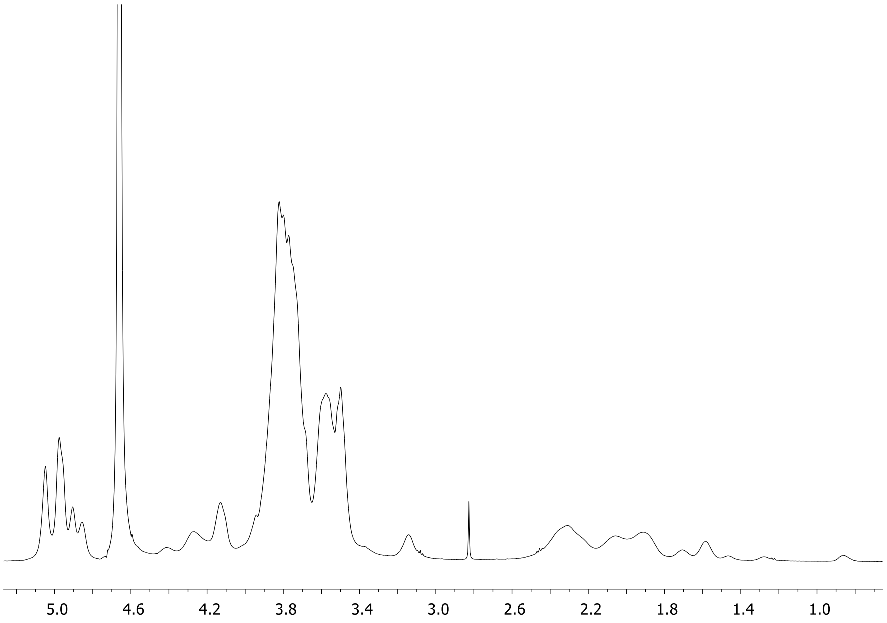

Figure S3. *^{1}H NMR spectrum of PGAβCyDArg2 (D_2O, 500 MHz)*

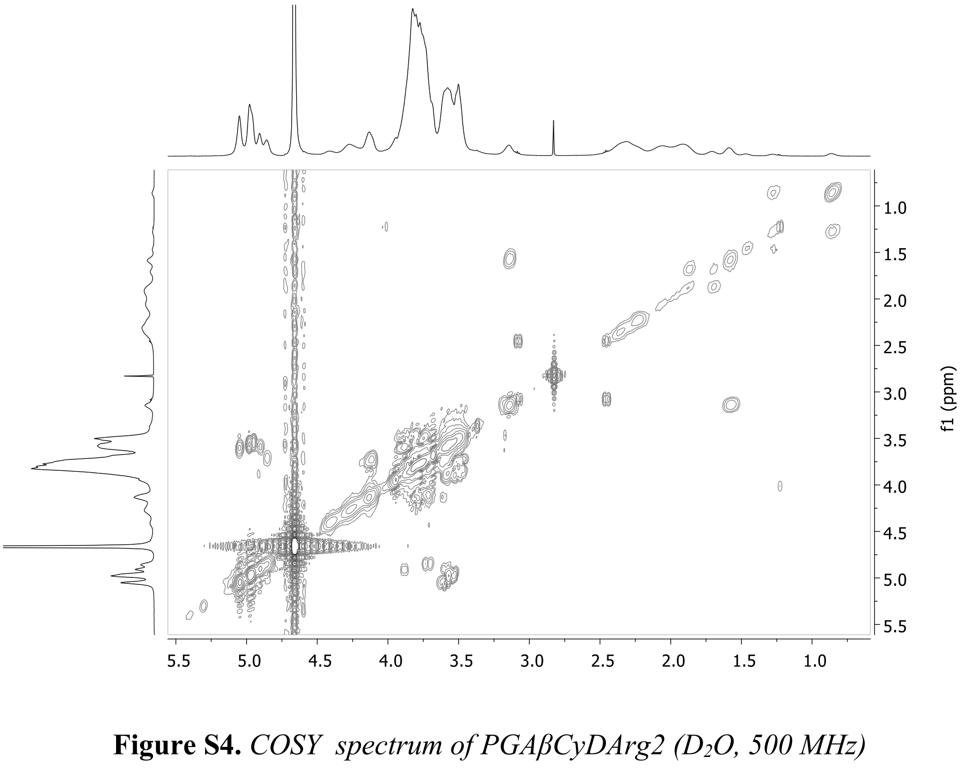

Figure S4. *COSY spectrum of PGAβCyDArg2 (D_2O, 500 MHz)*

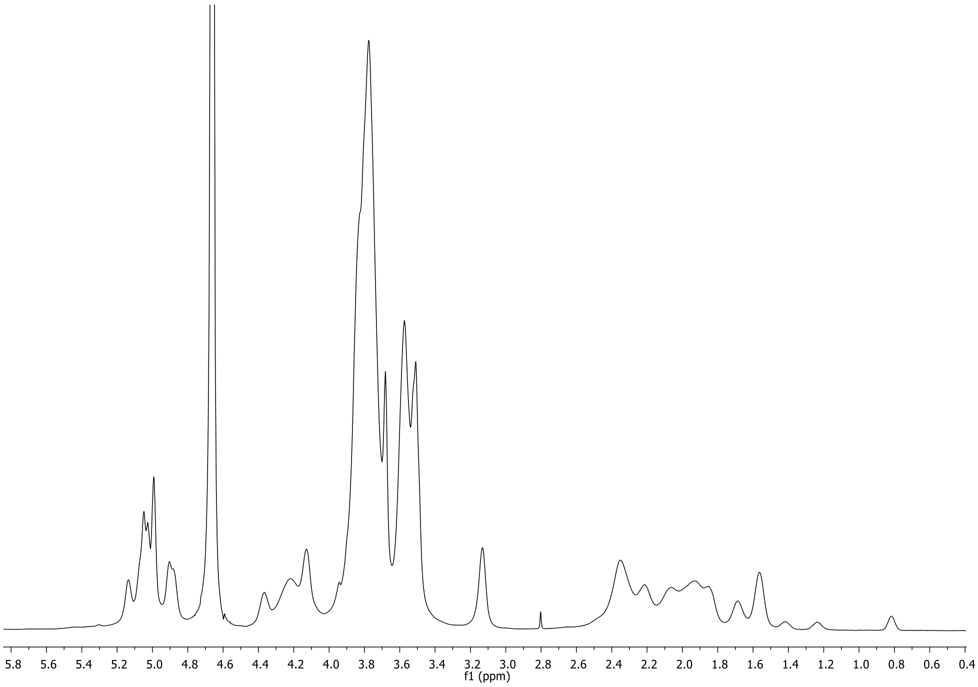

Figure S5. *^{1}H NMR spectrum of PGAγCyDArg3 (D_2O, 500 MHz)*

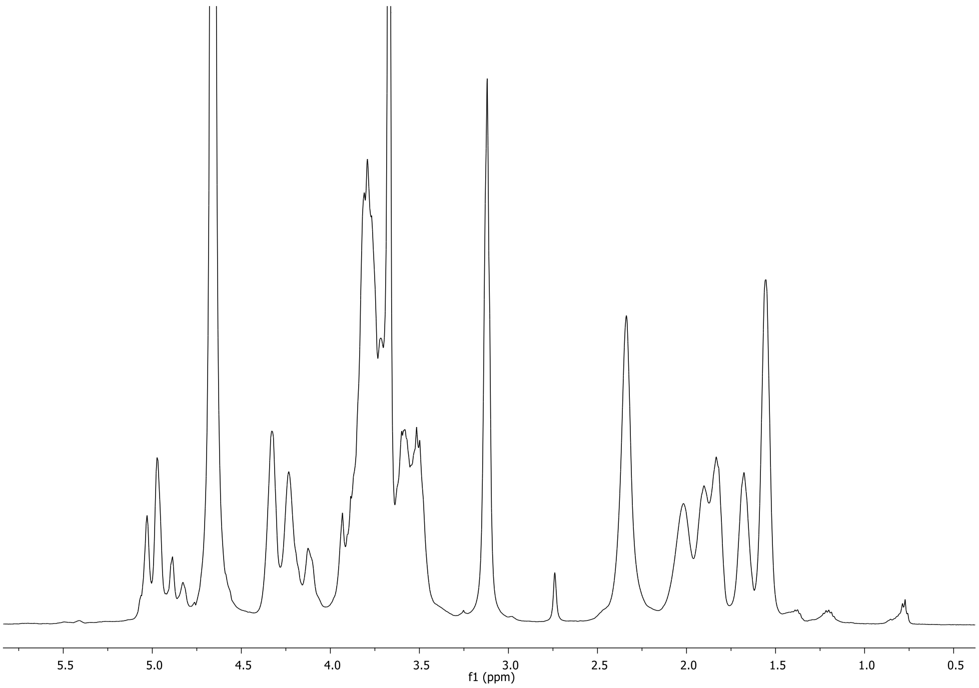

Figure S6. *^{1}H NMR spectrum of PGAβCyDArg4 (D_2O, 500 MHz)*

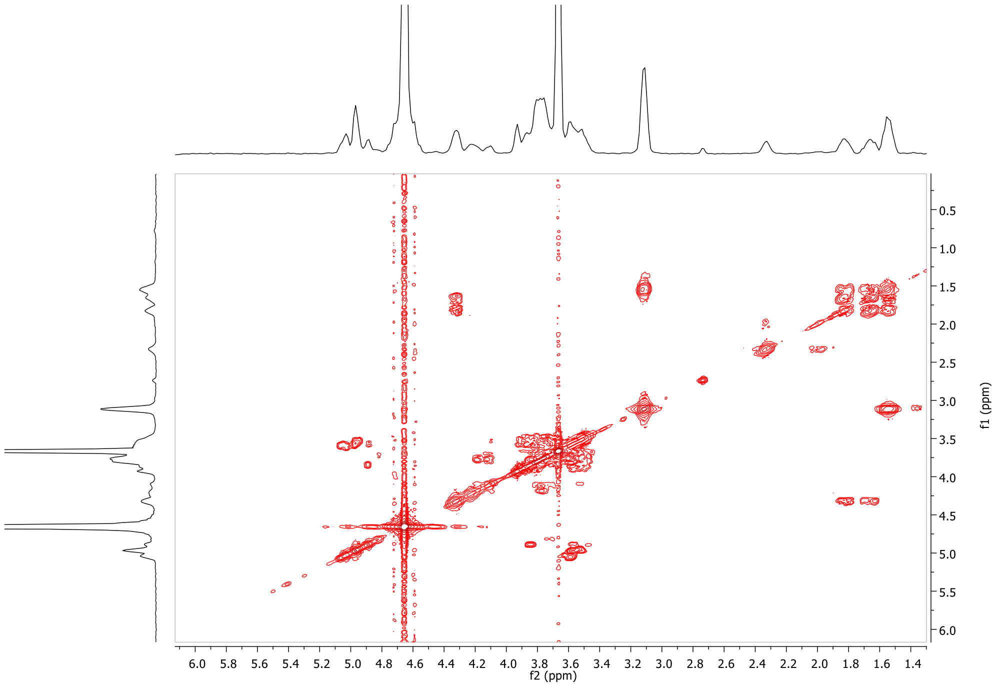

Figure S7. *COSY spectrum of PGAβCyDArg4 (D_2O, 500 MHz)*

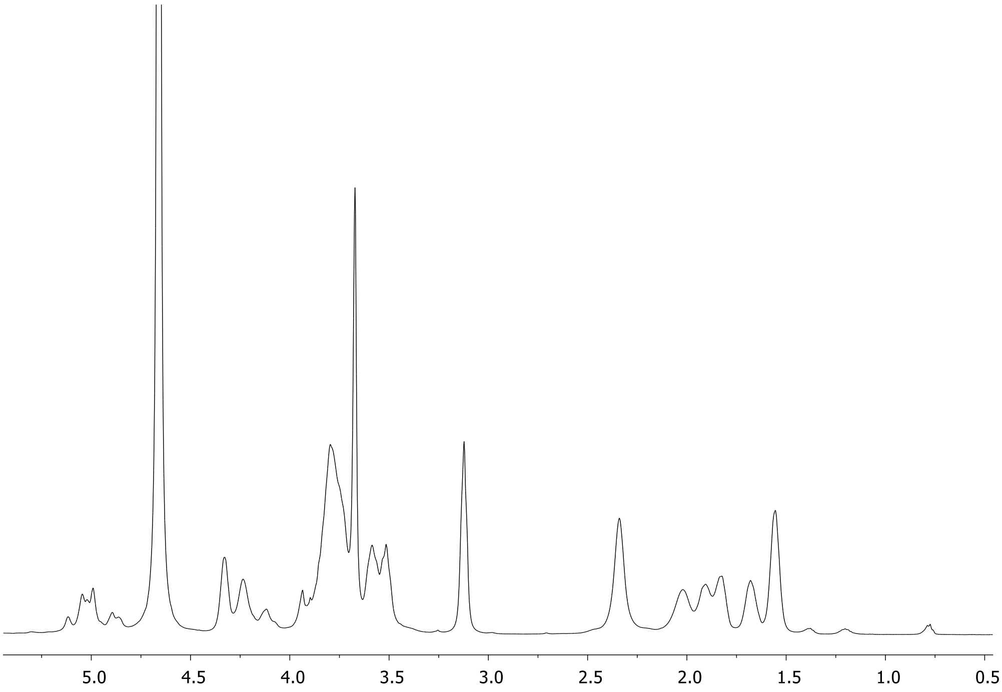

Figure S8. *^{1}H NMR spectrum of PGAγCyDArg5 (D_2O, 500 MHz)*

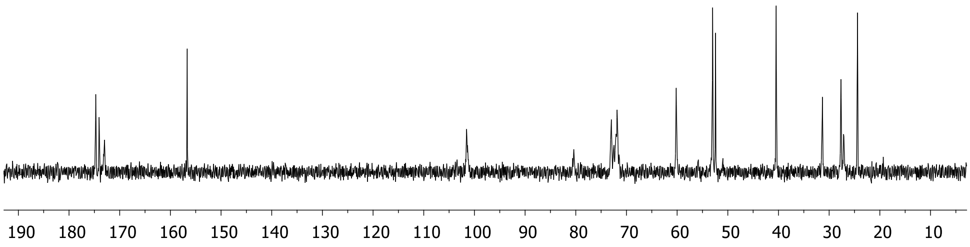

Figure S9. *^{13}C NMR spectrum of PGAγCyDArg5 (D_2O, 125 MHz)*

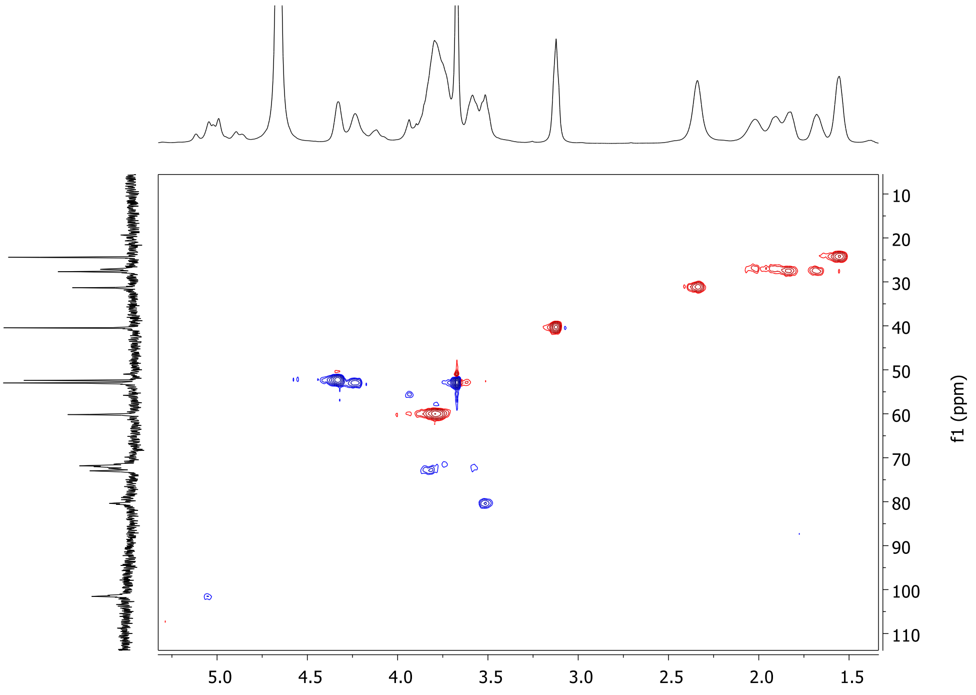

Figure S10. *HSQC spectrum of PGAγCyDArg5 (D_2O, 500 MHz)*

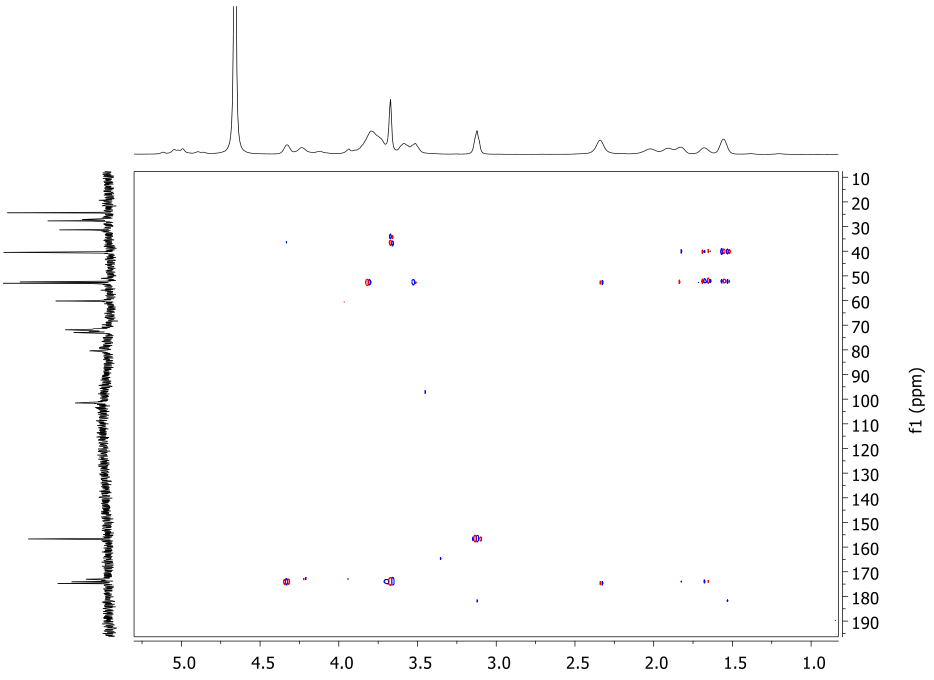

Figure S11. *HMBC spectrum of PGAγCyDArg5 (D_2O, 500 MHz)*

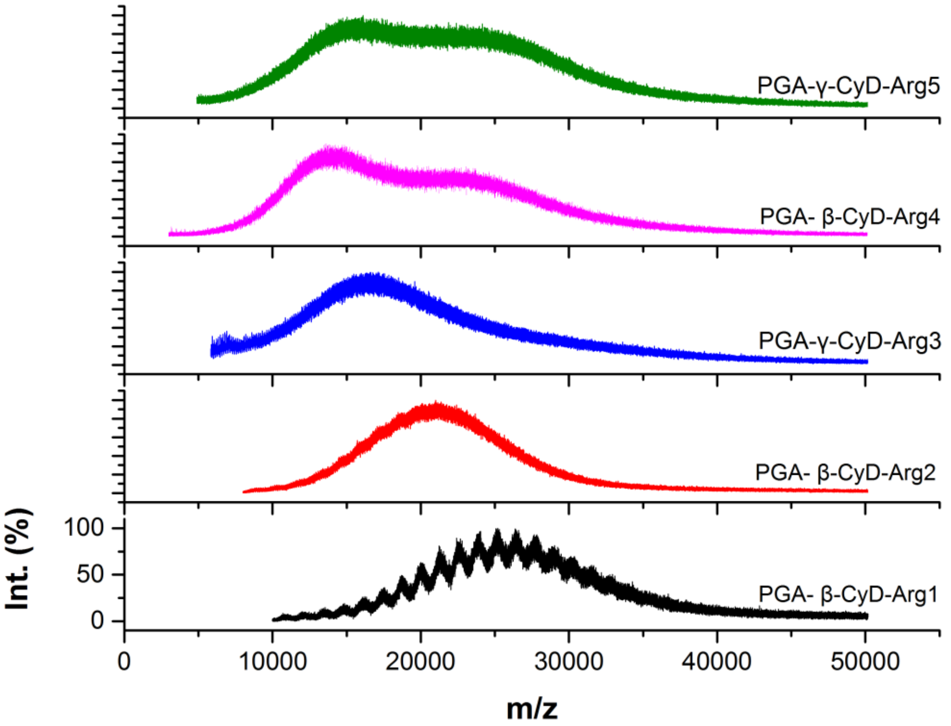

Figure S12. MALDI-TOF MS spectra of the PGA-β-CyD-Arg polymers

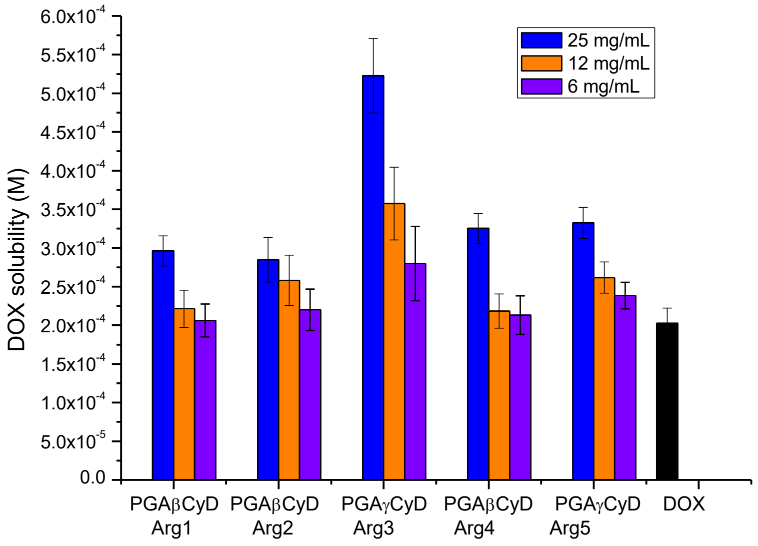

Figure 13. Solubility of DOX in the presence of polymers (phosphate buffer, 7.4 pH)
